# Supplementary material for: Peripheral intravenous catheter insertion and use of ultrasound in patients with difficult intravenous access: Australian patient and practitioner perspectives to inform future implementation strategies
Source: PLoS One. 2022 Jun 24;17(6):e0269788. doi: 10.1371/journal.pone.0269788 (PMC9231778; doi:10.1371/journal.pone.0269788)
Supplement: Attachment — Submitted filename: Response to reviewers feedback.docx [file pone.0269788.s002.docx]

Response to reviewers’ comments.

Re: PONE-D-21-22073. *Peripheral intravenous catheter insertion and use of ultrasound in patients with difficult intravenous access: Patient and practitioner perspectives to inform future implementation strategies.*

Thank you for your review of the above manuscript, please find responses to points raised below.

Overall, we would like to point out the lack of feedback and the stark contrast between the positive reviewers of reviewer one and the at times unprofessional review provided by reviewer 2. We request a third review and different academic editor.

Thank you for your consideration.

| **Comment** | **Response** |
| --- | --- |
| Reviewer 1 |  |
| The manuscript is technically sound. Survey was formed and preformed using a systematic approach. The thematic analysis themes that emerged are common in what I see in my practice. The statistical analysis consisted of some percentages of the respondents and that is all that was needed. Complying the themes that emerged does not call for a p-value. The manuscript was written in an intelligent manor and easy to follow. | Thank you, we would like to highlight the vast difference in feedback from reviewer 1 compared with reviewer 2 yet there was no explanation provided by the editor and we believe the emotive and unprofessional language provided by reviewer two has influenced the decision of the academic editor. |
| Reviewer 2 |  |
| This is a very confused paper with several bias: | We respectfully disagree and have concerns regarding the reviewer’s experience and knowledge of reported methods |
| It reports an experience that - even if true - should be referred exclusively to Australia (and this aspect is not stated, either in the title or in the abstract | We find the comment – *even if true*- to be disrespectful and not in keeping with the principles of GCP and the purpose of constructive and fair peer review. We are also disappointed this comment was not filtered by the academic editor and therefore request a different editor to handle to appeal.  However, we have made a change to the title to reflect this concern.  ‘Peripheral intravenous catheter insertion and use of ultrasound in patients with difficult intravenous access: Australian patient and practitioner perspectives to inform future implementation strategies’.  We note however that PLOS One publishes national studies as evidenced through recent publications of the Hungary experience in outpatient care and the Ghana experience of women using reproductive technology. |
| the selection of healthcare operators and of patients is largely arbitrary; it is likely that different sampling might have yielded different answers | This is a common limitation of opt in sampling. One we acknowledge in the limitations of the paper. However, we used a wide, rigorous sampling strategy one which we discuss in detail in the methods. We did not select participants we offered the interviews to patients and healthcare providers nationally. |
| it is not known (or not declared) whether all nurses and all patients answering the interview had a clear idea of what 'DIVA' means | The eligibility criteria for interviewees was experience of history with DIVA. This was thoroughly explored and explained during the information and consent process to ensure informed consent and patient eligibility requirements. This comment could be said for any intervention/healthcare experience is an unfair/unsupported statement with the description provided in the methods. |
| while a questionnaire might be somehow objective (sometimes) a direct interview leaves much room to non-scientific interpretation of the subject's answers; in other words, it is impossible to state in which degree the answers were 'directed' by the interviewer | This is an accepted, expected and routinely reported instrument (interview guide) which is considered best practice in qualitative methods which use interviewing. These methods use an interview guide to standardise questions and reduce bias – a process widely published across disciplines including nursing, medicine, psychology and more. Further the rigour of the study would be reduced without this instrument. |
| the overall impression is that this manuscript has few characteristics of a scientific paper; while the topic is obviously important and interesting, the way is addressed and discussed is very 'journalistic' and more apt to be published on a popular magazine than on a scientific journal | We firmly and wholeheartedly disagree with the reviewer, and in fact are disappointed that this feedback was included by the editor in our collated feedback.  We believe this viewpoint is biased towards quantitative methods and in direction contrast to PLOS One publishing and review policies.  We would direct you to other manuscripts which use the same methods, reporting framework and tone – which have been previously published in PLOS One and have been well cited and used internationally.  It is important to note the expert methodologist on paper one is the expert methodologist, senior academic and key writer of this manuscript.   - Not "just" an intravenous line: Consumer perspectives on peripheral intravenous cannulation (PIVC). An international cross-sectional survey of 25 countries - Experiences of women undergoing assisted reproductive technology in Ghana: A qualitative analysis of their experiences - Eliciting preferences for outpatient care experiences in Hungary: A discrete choice experiment with a national representative sample |
| Overall ratings |  |
| 1. Is the manuscript technically sound, and do the data support the conclusions?  The manuscript must describe a technically sound piece of scientific research with data that supports the conclusions. Experiments must have been conducted rigorously, with appropriate controls, replication, and sample sizes. The conclusions must be drawn appropriately based on the data presented.  Reviewer #1: Yes  Reviewer #2: No | Again, we would like to highlight the vast difference in feedback from reviewer 1 compared with reviewer 2.  The methods are rigorous conducted using best practice for qualitative methods and reported inline with internationally standardised and accepted reporting frameworks.  We believe this rating is reflective of reviewer 2 inexperience and lack of understanding of qualitative methods.  As such we request a third review. |
| 4. Is the manuscript presented in an intelligible fashion and written in standard English?  PLOS ONE does not copyedit accepted manuscripts, so the language in submitted articles must be clear, correct, and unambiguous. Any typographical or grammatical errors should be corrected at revision, so please note any specific errors here.  Reviewer #1: Yes  Reviewer #2: N | Again, we would like to highlight the vast difference in feedback from reviewer 1 compared with reviewer 2 |
