# Supplementary material for: Peripheral intravenous catheter insertion and use of ultrasound in patients with difficult intravenous access: Australian patient and practitioner perspectives to inform future implementation strategies
Source: PLoS One. 2022 Jun 24;17(6):e0269788. doi: 10.1371/journal.pone.0269788 (PMC9231778; doi:10.1371/journal.pone.0269788)
Supplement: Attachment — Submitted filename: PLOS Response to Review and Response.docx [file pone.0269788.s004.docx]

Thank you for the opportunity to revise the manuscript

Please see attached comments in relation to reviewer feedback for the manuscript PONE-D-21-22073R1

Response to Review and Response - Reviewer 1

The following comments are my observations according to the PLOS’s Criteria for Publication.

**1. The study presents the results of original research**

The original content of this research is in the approach. The researchers included both health care providers and patients during the survey. Much of the research conducted around ultrasound use and difficult intravenous access patients are quantitative including number of attempts, failure rates and dwell times. This original research is important because it takes us as readers to the patient experience as well as supporting the difficulties shared by health care workers when approaching venous depleted patient

When studying the experience of either the provider or the patient, the answers are subjective and are not confined to a specific criteria.

*Response*: Thank you no change made.

## 2. Results reported have not been published elsewhere

I have not seen research with this design specifically with difficult venous access patients.

*Response*: Thank you no change made.

## 3. Experiments, statistics, and other analyses are performed to a high technical standard and are described in sufficient detail.

## Using the Behavioral Change Wheel to perform a thematic analysis is a systematic method of interpreting the subjective answers given by health care providers and patients. The Behavioral Change Wheel is described in the text, but a visualization of this would help the reader understand more preciously. The thematic analysis is subjective interpretation process. The researchers gave examples of each theme to help the reader understand the themes. It is not necessary for every comment to be listed. The reader should understand the themes are filtered by the researchers own experience and bias. This is a qualitative study about patient and HCP experiences that requires interpretation not an easily measured outcome like failure.

*Response*: Thank you intended change to come – permission requested to include picture (Behaviour change wheel) within the manuscript awaiting copyright permission Michie et al.

**4. Conclusions are presented in an appropriate fashion and are supported by the data.**

It is clear to me from the manuscript that the patients have a poor healthcare experience, there is poor standardization of DIVA assessment that contributes to an ineffective escalation process. Theme one, harmful patient experiences, illustrated how the challenge of difficult intravenous access patients affect the patient experience. The theme “have a go” and “has a few goes and then comes to find me and gets me to do it” is common at the bedside. The clinician is encouraged to stick the patient even when they are not confident about placing the device. The conclusions are presented in an appropriate fashion and are supported by the answers given by the participants.

*Response*: Thank you no change made.

## 5. The article is presented in an intelligible fashion and is written in standard English.

## No issue.

*Response*: Thank you no change made.

## 6. The research meets all applicable standards for the ethics of experimentation and research integrity.

## The study was approved by the appropriate board.

*Response*: Thank you no change made.

## 7. The article adheres to appropriate reporting guidelines and community standards for data availability.

## There are no specific guidelines for collecting subjective survey information. the researchers chose a method to organize the answers and applied it to a framework. This is the best that can be done with this qualitative design. There are no quantitative measurements that can be measured against benchmarking.

## I stand by my original recommendation this research should be published.

## Matt Gibson

*Response*: Thank you no change made.

Response to Review and Response - Reviewer 2

**1. Patient interview size was small. How did you decide on the total n? Is this enough to map the themes?**

- refer to reporting guidelines

*Response*: No change made. Reference to reporting guidelines has been made in text. Please see the following statement contained on page 6, line 144-145

Results are reported in line with the Consolidated criteria for reporting qualitative research (COREQ) guidance.

**- appropriate framework for reporting qual work, sample size not defined apriori’**

*Response*: Thank you. This is a very large sample for qualitative work (n=78). Sample size was not defined apriori as recommended for these methods. Instead data saturation is used to determine when an appropriate sample size has been achieved. Therefore the sample size was appropriate to map themes.

**2. Mapping against COM-B sources of behaviour was not explained thoroughly. What exactly is COM-B and were does it come from? I believe C=Compatibility, O=Opportunity, M=Motivation, but what is B?**

COM-B referenced – contemporary reference.

*Response*: Thank you for your comment. The COM-B reference is a seminal framework and therefore cited in this paper. Since its publication the COM-B has been cited more than 6000 times in research articles (google scholar 02/2022). It is a useful and appropriate framework to situate the research. The COM-B is referenced for readers interested in learning more about the application of the framework.
